# Wandering Mucosal Melanoma Presenting as Occult Gastrointestinal Blood Loss Anemia

**DOI:** 10.7759/cureus.25614

**Published:** 2022-06-02

**Authors:** Aimen Farooq, Hamaad Rahman, Baha Aldeen Bani Fawwaz, Abu Hurairah

**Affiliations:** 1 Internal Medicine, AdventHealth Orlando, Orlando, USA; 2 College of Osteopathic Medicine, Kansas City University of Medicine and Biosciences, Kansas, USA; 3 Gastroenterology and Hepatology, AdventHealth Orlando, Orlando, USA

**Keywords:** iron deficiency anemia (ida), chronic gastrointestinal bleeding, endoscopic diagnosis, cancer immunotherapy, mucosal malignant melanoma, primary gastric malignant melanoma

## Abstract

Malignant melanoma is a highly aggressive cancer arising from the skin, retina, and mucosal lining of the respiratory, gastrointestinal (GI), or genitourinary tracts, all of which contain melanocytes. Mucosal or extracutaneous melanomas (ECMs) are rare accounting for 1% of all melanomas. We herein report a case of a metastatic mucosal melanoma presenting as occult blood loss anemia.

A 58-year-old male presented with generalized weakness, anorexia, weight loss, and intermittent melena for one year. On exam, he was tachycardic, borderline hypotensive, and pale without epigastric tenderness. Labs showed severe anemia [hemoglobin, Hgb 3.8 mg/dL, mean corpuscular volume (MCV) 72 fl] for which he received two units of red cells. Endoscopy revealed an 8 mm non-bleeding, gastric ulcer with a raised border and a clean base on the wall of the gastric body. Histologic analysis was consistent with malignant melanoma displaying strong positivity for S-100, Melan A, and HMB 45 stains. The CT of the abdomen revealed multifocal metastatic disease with subcutaneous, intramuscular, and perinephric implants with suspicion of small bowel carcinomatosis. The patient underwent an excisional biopsy for the abdominal wall mass and surgical pathology confirmed melanoma. The patient is planned to be started on immunotherapy for advanced disease.

Most melanomas found in the GI tract are metastatic. Mucosal melanoma presenting as a gastric ulcer is extremely rare. As a result, metastasis from other sites must be ruled out before making a diagnosis of primary gastric melanoma (PGM). In our case, a widespread disease with unknown primary elucidated the diagnosis but post-operative inspection failed to find any potential lesion on the skin, genitals, or other organs, suggesting the possible diagnosis of metastatic gastric melanoma. However, follow-up is still required to confirm the diagnosis according to the established criteria. Pathologic diagnosis of melanoma requires the identification of melanin in the cytoplasm and immunohistochemistry with specific markers such as S-100, Melan A, and HMB-45. Although the pathologic diagnosis of PGM is similar to cutaneous melanoma, preoperative diagnosis is difficult due to the extremely low incidence, lack of obvious melanin pigmentation, similar microscopic patterns as more common gastric cancers, and lack of awareness among physicians and pathologists.

The prognosis of mucosal melanoma is poor, with a five-year survival rate of 25% versus 80% for cutaneous melanoma. Advanced age, surgically unresectable disease, and lymph node involvement are all poor prognostic markers. There is no standard protocol for treatment. Surgery is the only curative treatment for the resectable disease. Adjuvant chemotherapy, radiation, and immunotherapy have an established role in cutaneous melanoma but there is only limited data on adjuvant systemic therapy with mucosal melanoma. Further research is imperative to establish proper management guidelines for this rare disease entity.

## Introduction

Malignant melanoma is a highly aggressive cancer arising from the skin, retina, and mucosal lining of the respiratory, gastrointestinal (GI), or genitourinary tracts, all of which contain melanocytes [1]. Mucosal or extracutaneous melanomas (ECMs) are rare accounting for 0.3% of all cancer diagnoses and ~1% of all melanomas [2-3]. The most common sites for mucosal melanomas are head and neck in men and vulvovaginal in women [4-5]. Although cutaneous melanomas frequently metastasize to the GI tract, primary GI melanomas, especially gastric melanoma, are extremely rare [6-7]. Mucosal melanomas are interesting due to their aggressive nature and poor prognosis when compared to their cutaneous counterparts. We herein report a case of mucosal melanoma with probable gastric origin presenting as occult blood loss anemia.

## Case presentation

A 58-year-old male with a past medical history of hypertension, type 2 diabetes, and hypothyroidism presented to the emergency department (ED) with complaints of weakness, fatigue, and shortness of breath over the past few months. He endorsed intermittent black, tarry stools, dizziness, loss of appetite, and weight for the past year. He reports noticing enlarging masses in the right upper quadrant of his abdomen and right axillary area over the past few weeks which concerned him since his friend had similar symptoms and had been diagnosed with lymphoma in the past. He denied pertinent surgical or family history. He never had a colonoscopy or esophagogastroduodenoscopy (EGD) in the past. Upon arrival, the patient was tachycardic (heart rate, HR 110 beats per minute) otherwise hemodynamically stable. Physical exam was notable for a 5-cm firm, non-fluctuating, and non-tender subcutaneous mass in the right upper quadrant of the abdomen, as well as a 5-cm palpable, firm, mobile, non-tender, and non-fluctuating lymph node in the right axillary region. 

Laboratory testing (Table 1) was consistent with severe iron deficiency anemia and reactive thrombocytosis for which he received two units of packed red blood cells (pRBCs).

**Table 1:** Pertinent laboratory work-up results. Hgb, hemoglobin; MCV, mean corpuscular volume; TIBC, total iron binding capacity

Laboratory test	Observed value	Reference range
White blood cells	8.66 10*3/uL	3.4 - 9.6 10*3/uL
Hgb	3.8 g/dL	13.2 - 16.6 g/dL
Hematocrit	14.4 %	38.3% - 48.6%
MCV	72 fL	80 - 100 fL
Platelets	499 10*3/uL	135 - 317 10*3/uL
Iron	13 ug/dL	45 - 182 ug/dL
Ferritin	5 ng/mL	24 - 336 ng/mL
TIBC	532 ug/dL	221 - 481 ug/dL
Iron saturation %	2%	30% - 44%
Transferrin	380 mg/dL	200 - 360 mg/dL

**Figure 1:**
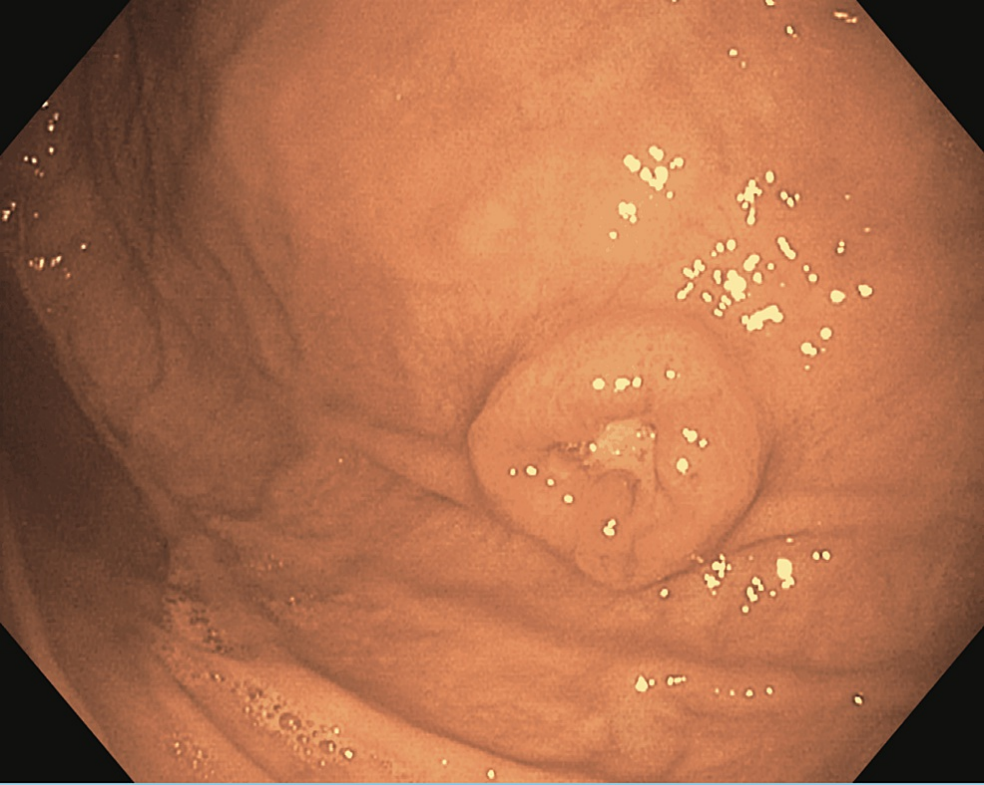
Endoscopic image of an 8-mm non-bleeding, cratered ulcer with a clean base on the anterior wall of the gastric body (Forrest Class III).

An EGD demonstrated an 8-mm, non-bleeding, clean base gastric ulcer with raised borders (Forrest Class III) on the anterior wall of the gastric body (Figure 1).

**Figure 2:**
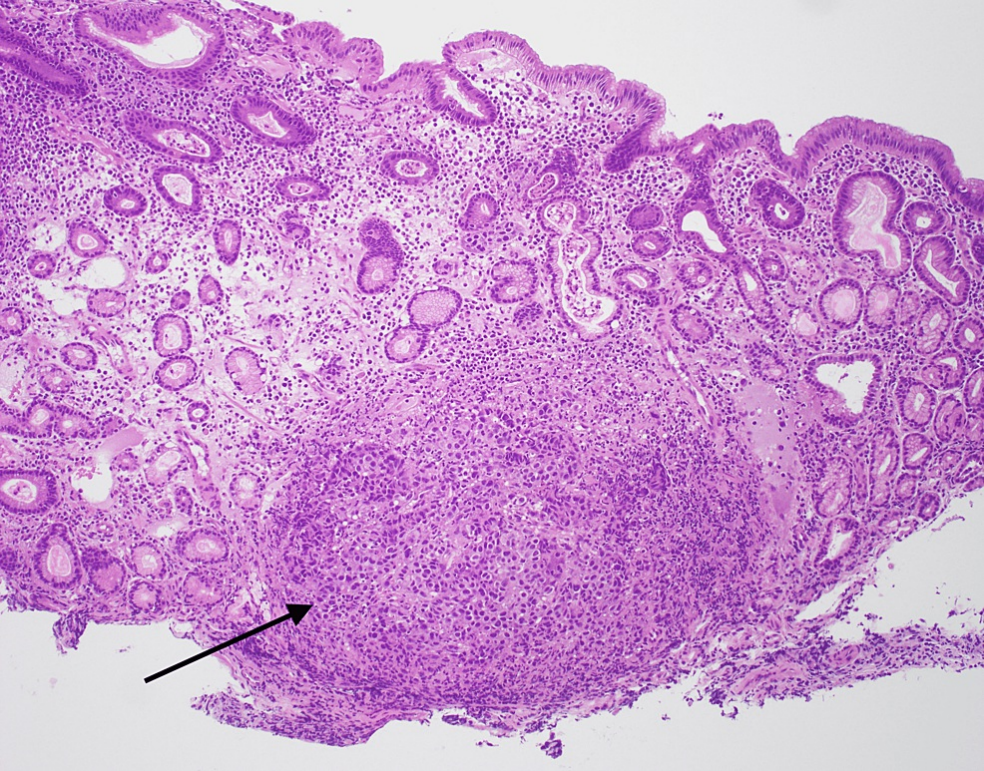
Endoscopic biopsy of the stomach ulcer: pathology showing nodular melanoma cells in a nodular pattern (black arrow) and surrounding normal gastric epithelium.

**Figure 3:**
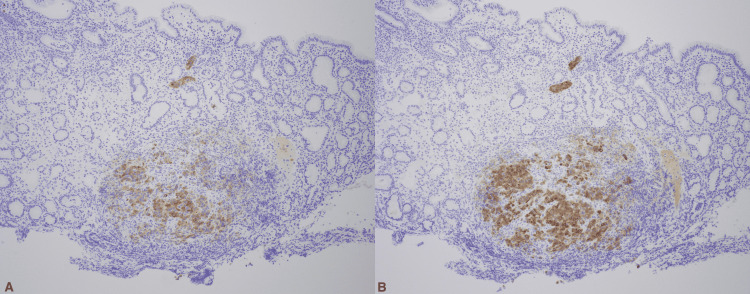
Immunohistochemical staining showing A) positive Melan A in melanoma cells and negative in stomach epithelium and B) positive HMB 45 in melanoma cells and negative in stomach epithelium.

Histopathological analysis showed an aggregate of malignant tumor cells in a nodular pattern (Figure 2) which displayed strong positivity with S-100 protein, HMB-45, and Melan-A, consistent with malignant melanoma (Figure 3a-b).

**Figure 4:**
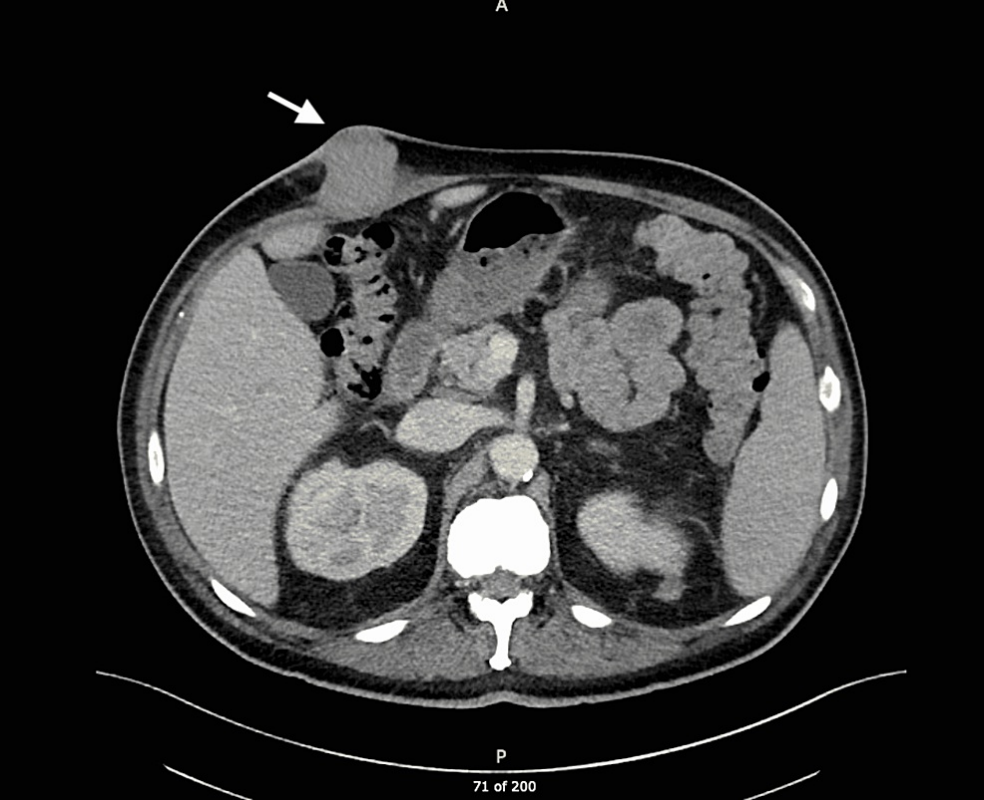
Axial CT image showing dominant lesion (arrow) in the right upper abdominal wall.

A contrast CT scan of the abdomen which was performed for staging revealed multiple subcutaneous and intramuscular implants within the abdomen and pelvis including a dominant lesion in the right upper abdominal wall measuring 4.3 cm x 4.3 cm, bilateral perinephric tumor implants, periportal and gastrohepatic ligament adenopathy, multiple areas of thickening and nodularity in the small bowel suspicious for carcinomatosis, and mild mesenteric edema. A CT angiogram of the chest showed right paratracheal lymphadenopathy and a 6.6-mm medial right apical pulmonary nodule (Figure 4).

The patient underwent an excisional biopsy for the abdominal wall mass, consistent with metastatic melanoma, positive for S-100 protein and Melan-A, and negative for cytokeratin AE1/AE3. Detailed inspection failed to find any potential lesions on the skin, eyes, or genitals. The diagnosis of metastatic mucosal melanoma was established, and the patient was discharged with plans to start combination immunotherapy with Nivolumab and Ipilimumab. Currently, the patient is continuing treatment with immunotherapy.

## Discussion

Melanomas arise from the malignant transformation of melanocytes, commonly in cutaneous tissues and infrequently in mucosal tissues including the GI, respiratory, and genitourinary tracts. Approximately 1.4% of all melanomas arise from mucosal tissues and have shown higher rates among women compared to men [8-9]. Mucosal melanomas are more aggressive and carry a worse prognosis than their cutaneous counterparts, making accurate and early diagnosis critically important [1]. 

Due to the rare nature of mucosal melanomas, information regarding their pathogenesis is limited. Cutaneous melanomas usually develop because of mutations in the BRAF gene, whereas studies have shown that 16%-39% of mucosal melanomas develop due to mutations in the KIT gene. Unlike the role of ultraviolet light exposure in cutaneous melanomas, there are no identifiable risk factors for mucosal melanomas. 

**Figure 5:**
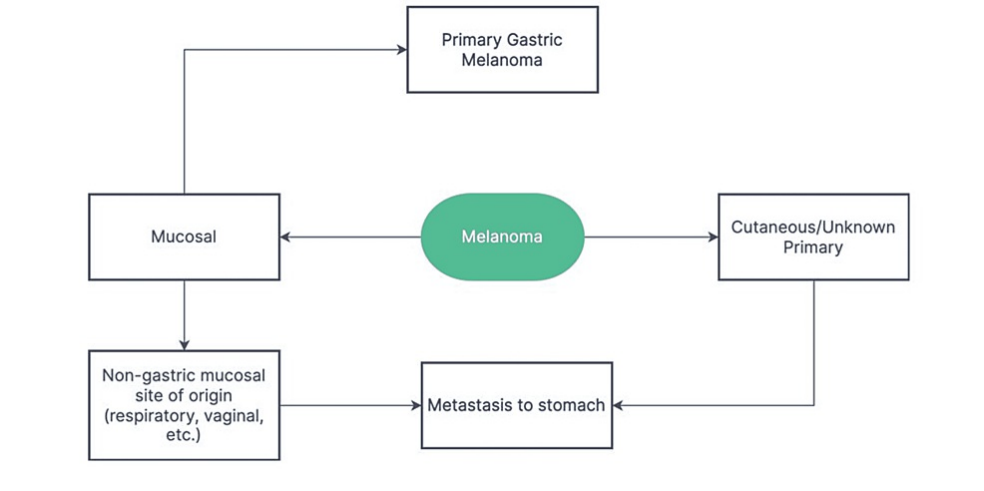
Schematic representation of different types of melanomas found in the stomach.

Mucosal melanomas of the GI tract can either be primary or secondary/metastatic (Figure 5). Primary gastric melanomas (PGMs) make up less than 1% of all ECMs [6-7]. Therefore, before diagnosing a primary lesion of the GI tract, it is imperative to perform a thorough cutaneous and ophthalmologic examination to rule out possible primary cutaneous or ocular lesions. 

Given the rarity of the disease, there are no agreed-upon diagnostic criteria for PGMs, rendering it hard to distinguish metastatic gastric melanoma from an advanced PGM with metastatic disease, as in our case. Blecker et al. suggested the following criteria for diagnosing PGM: 1. absence of invasive or in situ cutaneous melanocytic lesions and 2. in situ changes in the GI epithelium [10]. An alternative approach by Song et al. suggested the following: 1. a single lesion of melanoma in the stomach proven by pathology; 2. absence of cutaneous lesions anywhere else in the body; 3. no history of melanoma; and 4. disease-free survival of at least 12 months after curative surgery [11]. 

The proposed diagnostic criteria suggest a lack of metastatic lesions as a criterion for diagnosis, however, a PGM diagnosed in the later stages could very well present with a gastric lesion as well as concomitant metastatic lesions. In a systematic review by Mellotte et al., 10 out of 34 patients with PGM were found to have distant solid metastasis in organs including the liver, brain, and lungs at initial presentation [9]. Additionally, the 12-month survival rate following treatment has been shown to vary significantly, making it an unreliable criterion for diagnosis. 

In our case, a thorough full-body cutaneous examination and ophthalmologic inspection did not reveal any other potential primary lesions. Furthermore, a colonoscopy was also performed which did not reveal any potential primary lesions. The patient did not have any history of melanoma. Although the patient did have significant metastasis throughout this body, it is quite possible that this has spread from the primary gastric lesion. 

Similarly, there are no clear staging guidelines for PGMs. The American Committee on Cancer (AJCC) guidelines for head and neck mucosal melanomas are normally improvised for the staging of GI mucosal melanomas [1]. Patients with PGM can experience several non-specific symptoms including abdominal pain, weight loss, occult upper GI bleed, and anemia. Because of a lack of specificity in clinical presentation, physicians often suspect other more common gastric cancers before considering PGM [1, 9, 11]. 

The CT scan is an acceptable choice for imaging and commonly reveals thickening of the gastric wall. EGD with a biopsy of the lesion plays a pivotal role in diagnosis. The majority of documented cases of PGM had large ulcerated or polypoidal masses of varying size in the stomach. Oftentimes lesions will be pigmented, however, lesions can also be amelanotic, so immunohistochemistry staining becomes critical for establishing the diagnosis [11]. 

In localized cases, total resection of the lesion with negative margins has proven to be curative. In metastatic cases, other treatment modalities should be considered as surgical resection is often not a viable option. Chemotherapy is not an effective treatment option against melanoma due to its resistance and low response rate [9]. Novel therapies focusing on molecular targets, such as anti-PD-1 agents and cytotoxic T-lymphocyte antigen-4 checkpoint inhibitors, have shown to be the most effective treatment [12]. Currently, the Food and Drug Administration (FDA) has approved ipilimumab, a cytotoxic T-lymphocyte antigen-4 checkpoint inhibitor, and nivolumab, an anti-PD-1 agent, for treating mucosal melanomas. Recent studies suggest that these two agents used in combination showed greater efficacy than either agent alone [13]. 

Overall PGM has a poor prognosis, with a one-year survival rate of less than 60% and a five-year survival rate of less than 3% [9, 11]. The most important prognostic factor is the presence of metastatic lesions, as patients with metastasis had significantly lower survival rates [9]. This further emphasizes the importance of raising awareness regarding the need for early detection of the disease which can significantly improve outcomes by stopping the spread of the disease.

## Conclusions

Malignant melanoma, although thought to be a primary cutaneous disease, can arise from mucosal tissues. The rarity of gastric melanomas, non-specific presenting symptoms, amelanotic lesions on the endoscopic exam, and the lack of clear guidelines for diagnosing a PGM, make diagnosis extremely challenging. Cutaneous and ocular lesions must be ruled out before the diagnosis of PGM is established. Early detection of the disease is imperative as metastasis is the most important prognostic factor. It is crucial to always consider immunohistochemistry staining. Surgery is the first-line treatment for localized disease, whereas immunotherapy has proven effective for metastatic disease. Further research is needed to establish diagnostic and management guidelines for this rare disease entity.

## References

[1] Mihajlovic M, Vlajkovic S, Jovanovic P, Stefanovic V (2012). Primary mucosal melanomas: a comprehensive review. Int J Clin Exp Pathol.

[2] Lourenço SV, Fernandes JD, Hsieh R, Coutinho-Camillo CM, Bologna S, Sangueza M, Nico MM (2014). Head and neck mucosal melanoma: a review. Am J Dermatopathol.

[3] Spencer KR, Mehnert JM (2016). Mucosal melanoma: epidemiology, biology and treatment. Cancer Treat Res.

[4] Patel SG, Prasad ML, Escrig M (2002). Primary mucosal malignant melanoma of the head and neck. Head Neck.

[5] Chang AE, Karnell LH, Menck HR (1998). The National Cancer Data Base report on cutaneous and noncutaneous melanoma: a summary of 84,836 cases from the past decade. The American College of Surgeons Commission on Cancer and the American Cancer Society. Cancer.

[6] Lam KY, Law S, Wong J (1999). Malignant melanoma of the oesophagus: clinicopathological features, lack of p53 expression and steroid receptors and a review of the literature. Eur J Surg Oncol.

[7] Lens M, Bataille V, Krivokapic Z (2009). Melanoma of the small intestine. Lancet.

[8] McLaughlin CC, Wu XC, Jemal A, Martin HJ, Roche LM, Chen VW (2005). Incidence of noncutaneous melanomas in the U.S. Cancer.

[9] Mellotte GS, Sabu D, O'Reilly M, McDermott R, O'Connor A, Ryan BM (2020). The challenge of primary gastric melanoma: a systematic review. Melanoma Manag.

[10] Blecker D, Abraham S, Furth EE, Kochman ML (1999). Melanoma in the gastrointestinal tract. Am J Gastroenterol.

[11] Song W, Liu F, Wang S, Shi H, He W, He Y (2014). Primary gastric malignant melanoma: challenge in preoperative diagnosis. Int J Clin Exp Pathol.

[12] Shoushtari AN, Munhoz RR, Kuk D (2016). The efficacy of anti-PD-1 agents in acral and mucosal melanoma. Cancer.

[13] D'Angelo SP, Larkin J, Sosman JA (2017). Efficacy and safety of Nivolumab alone or in combination with Ipilimumab in patients with mucosal melanoma: a pooled analysis. J Clin Oncol.

